# Experimental Behavior of Precast Bridge Deck Systems with Non-Proprietary UHPC Transverse Field Joints

**DOI:** 10.3390/ma14226964

**Published:** 2021-11-18

**Authors:** Mohamed Abokifa, Mohamed A. Moustafa

**Affiliations:** Department of Civil and Environmental Engineering, University of Nevada, Reno, NV 89557, USA; mabokifa@nevada.unr.edu

**Keywords:** ultra-high performance concrete, non-proprietary ultra-high performance concrete, precast bridge deck, field joints, accelerated bridge construction, full-scale testing, experimental behavior

## Abstract

Full-depth precast bridge decks are widely used to expedite bridge construction and enhance durability. These deck systems face the challenge that their durability and performance are usually dictated by the effectiveness of their field joints and closure joint materials. Hence, commercial ultra-high performance concrete (UHPC) products have gained popularity for use in such joints because of their superior mechanical properties. However, the proprietary and relatively expensive nature of the robust UHPC mixes may pose some limitations on their future implementation. For these reasons, many research agencies along with state departments of transportation sought their way to develop cheaper non-proprietary UHPC (NP-UHPC) mixes using locally supplied materials. The objective of this study is to demonstrate the full-scale application of the recently developed NP-UHPC mixes at the ABC-UTC (accelerated bridge construction university transportation center) in transverse field joints of precast bridge decks. This study included experimental testing of three full-scale precast bridge deck subassemblies with transverse NP-UHPC field joints under static vertical loading. The test parameters included NP-UHPC mixes with different steel fibers amount, different joint splice details, and joint widths. The results of this study were compared with the results of a similar proprietary UHPC reference specimen. The structural behavior of the test specimens was evaluated in terms of the load versus deflection, reinforcement and concrete strains, and full assessment of the field joint performance. The study showed that the proposed NP-UHPC mixes and field joint details can be efficiently used in the transverse deck field joints with comparable behavior to the proprietary UHPC joints. The study concluded that the proposed systems remained elastic under the target design service and ultimate loads. In addition, the study showed that the use of reinforcement loop splices enhanced the load distribution across the specimen’s cross-section.

## 1. Introduction

**Figure 1:**
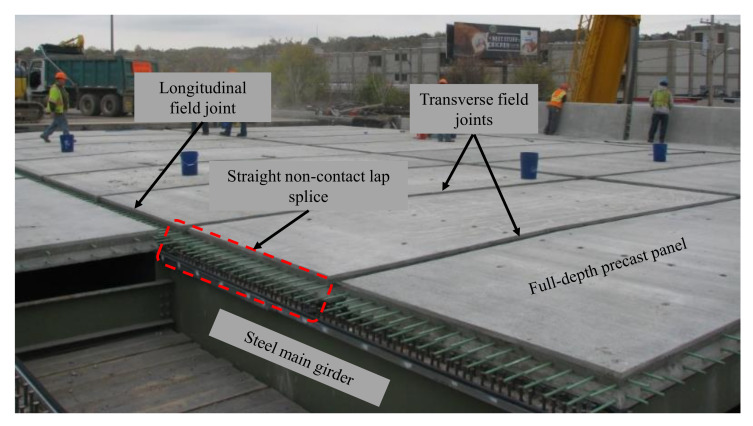
Types of field joints in a typical prefabricated bridge deck system.

Cast-in-place (CIP) construction techniques have been widely used for many years in the construction of bridge decks around the nation. The reason for the wide implementation of these CIP systems is the relatively cheaper costs relative to other systems and easier construction. However, these systems showed a lack of performance, degradation in strength and less durability after spending many years in service. As a result, nearly 56,000 US bridges are considered structurally deficient based on the records of the American Road and Transportation Builders Association (ARTBA) [1]. While bridge decks deteriorate faster than the other bridge components, more than $8 billion are spent annually on repairing or replacing these deteriorated decks [1]. Approximately 85% of the US daily commuters travel on state-owned bridges, which makes it more difficult to use the traditional construction techniques or CIP methods in the replacement or rehabilitation of the deteriorated decks. This has paved the way for a wider implementation of prefabricated construction techniques to accelerate the deck erection. Prefabricated bridge decks (PBES), which is one of the accelerated bridge construction (ABC) applications, can enhance constructability issues, offer higher quality, provide accelerated and safer construction, and minimize traffic disruption. On the other hand, the prefabricated bridge deck elements usually require to be connected on the field through field joints that could form a weak link that affects the overall system performance. These field joints can be classified into two main types. Transverse joints that run perpendicular to the traffic flow direction and longitudinal joints that run along the longitudinal axis of the bridge, i.e., parallel to the traffic direction. Figure 1 shows both field joint types in a typical precast bridge deck system. The use of traditional joint closure materials like conventional concrete and non-shrink grouts for such joints has resulted in either relatively wide joints because of the required longer development length, or narrow joints with mechanical splicing of reinforcement or post tensioning. Moreover, these types of joints require longer time and effort to fabricate than using advanced materials like UHPC, hence, they are not adequate as ABC techniques. Nonetheless, interface cracking under service and ultimate design load levels has been one of the main issues associated with the use of traditional materials in field joints. With the recent introduction of advanced materials to the bridge community, many researchers have explored many types of these advanced construction materials for use as a bridge deck field joint filler material. Some researchers focused on the experimental investigation and demonstration of the bridge decks with field joints filled with advanced grouts [2,3,4], high-performance concrete (HPC) [2,5], and HPC with fiber reinforcement [5]. However, many of these research efforts included special considerations for the reinforcement splices inside the joint, such as rebar post-tensioning, using of mechanical splices, using of headed bars, or adding rebar confinement inside the joint to decrease the required splice length.

Currently, ultra-high performance concrete (UHPC) has gained a great significance and reputation as a bridge deck joint material. Many research studies demonstrated that the ideal UHPC field joint has a diamond-shaped shear key, 15.2–20.3 cm joint width, and traditional straight or loop splices. These typical joint details are sufficient in transferring shear and bending across the prefabricated deck elements [6,7,8,9]. However, the use of UHPC comes with several challenges. First, the commercial UHPC products are costly and the material can be 15 to 20 times more expensive than conventional concrete. Moreover, commercial UHPC is proprietary and only supplied by a limited number of vendors. This often limits state DOTs (Department of Transportation) that are trying to avoid sole-sourcing among other bidding issues to use UHPC. Hence, there is a growing interest from various state DOTs and research agencies in developing non-proprietary UHPC (NP-UHPC) mixes to be used for different bridge applications. These research efforts aimed at making UHPC more accessible and less expensive through using locally available materials in the NP-UHPC mixtures [10,11,12,13,14,15,16]. The NP-UHPC is much cheaper than the P-UHPC as the typical P-UHPC and NP-UHPC costs are estimated to be around $3300 and $1300 per cubic meter, respectively [17,18]. However, there are still some limitations on the large-scale production of UHPC because of the long mixing time and the relatively small batch size. This has motivated researchers and precast concrete plants to explore different ways of mixing UHPC in large quantities [19,20]. A recent literature study showed that it is applicable to mix NP-UHPC mixtures in large quantities to fabricate a full UHPC pi girder bridge using the conventional ready mix-trucks [19]. One major contribution to this field of study is the recent research work done by the five consortium universities within the ABC university transportation center (ABC-UTC) in the US [20,21]. The University of Oklahoma has led this project by developing the mix design and material testing of the baseline NP-UHPC mix [22]. The information regarding the baseline mix design was shared with the other universities to examine the viability of using this NP-UHPC mix in various ABC applications. The experimental work covered in this paper, which was part of this wider collaboration project, was conducted by the University of Nevada, Reno (UNR). The main role for UNR in this project was to first extend the use of the NP-UHPC mix design to develop NP-UHPC mixes using locally available materials in Nevada (NV) and California (CA). Next, we conducted experimental testing of full-scale precast bridge deck panel systems with transverse and longitudinal field joints that were filled with the developed NP-UHPC mixes. This paper covers the experimental results of the transverse joint specimens only to provide a detailed discussion of the structural behavior and analysis of the joint performance.

The overall objective of this study is to investigate the structural performance of the prefabricated deck elements with NP-UHPC transverse filed joints. Moreover, the study aimed at validating the use of the NP-UHPC as a closure joint material in comparison with a robust commercial UHPC product. Other objectives of this study included engineering and optimization of the NP-UHPC mixes and joint details to provide efficiently equivalent systems at a cheaper cost. The engineering solutions used in this study included the optimization of the amount of steel fibers in the NP-UHPC mixes, varying the joint width, using different joint splices, and varying the distribution of the overlapped reinforcement. Three full-scale specimens were experimentally tested in this study under static vertical loading. The paper includes several sections that present a discussion of the development of the mix, results from a similar precedent study with commercial UHPC to use for reference, details of the experimental program, test results/discussion, and conclusions.

## 2. Background

This section provides detailed information about the development, composition and material characterization of the NP-UHPC mix utilized in this study. Moreover, this section also highlights the experimental test results of a similar bridge deck specimen with a proprietary UHPC (P-UHPC) transverse field joint previously tested by the authors [23,24]. The main test results obtained from that reference P-UHPC specimen are shown in this section to allow for comparisons with the NP-UHPC specimens tested in this study.

### 2.1. NP-UHPC Mix

Many state DOTs have commissioned university research teams to develop NP-UHPC mixes using locally sourced materials. In fact, this has resulted in the development of cheaper UHPC mixes with comparable mechanical characteristics to commercial UHPC mixes. One of these research efforts is the work done by the University of Oklahoma (OU) [22]. They developed an NP-UHPC mix with a 30% cement slag replacement by weight to reduce the overall cost of the material. This NP-UHPC mix was selected by the ABC university transportation center (ABC-UTC) for further experimental testing in different ABC applications, such as the bridge deck field joints. UNR was part of this wide research project and our role was to experimentally test the same NP-UHPC mix using locally available materials in the NV and CA states in the bridge deck field joints. Our work at UNR comprised three main parts which included first the development and material testing of different NP-UHPC mixes to engineer and optimize the materials and the amount of the steel fibers needed inside the mix [25]. As a result of this preliminary assessment, two NP-UHPC mixes were proposed and selected for further experimental testing and implementation in deck field joints. The large-scale experimental testing of the NP-UHPC field joints was divided into two parts. The first part included full-scale testing of portions of deck bulb tee (DBT) girders with NP-UHPC longitudinal field joints [26]. The second part, which was presented in this paper, included full-scale testing of precast bridge deck assemblies with transverse NP-UHPC field joints. The last part of this project is what we present in detail in this paper. Two typical NP-UHPC mixes were used in this study that differed only in the percentage of the steel fibers included in the mix (i.e., 1% versus 2% by volume, which corresponds to 3% versus 6% by weight). The dimensions of the steel fibers are 13 mm in length and 0.2 mm in diameter with a nominal tensile strength of 2750 MPa. The mixing proportions and the material sources of both mixes are shown in Table 1.

As mentioned earlier, the main aim of the first step in this project was to determine the main mechanical properties of the developed NP-UHPC mixes. Hence, this paper does not present material characterization but provides an overview of the main mechanical properties of the NP-UHPC mixes used herein. For more details, the reader is referred to the additional information and test results and discussion reported in [25,26]. Table 2 shows the results of the 28-day compressive strength, flexural strength and direct tensile strength for both mixes. It is noted that both mixes reached the 97 MPa compressive strength limit after 7 Days, which is the threshold recommended by FHWA (Federal Highway Administration) to open the bridges for traffic or to strip off the formwork [27].

### 2.2. Reference Specimen with P-UHPC Joint

**Figure 2:**
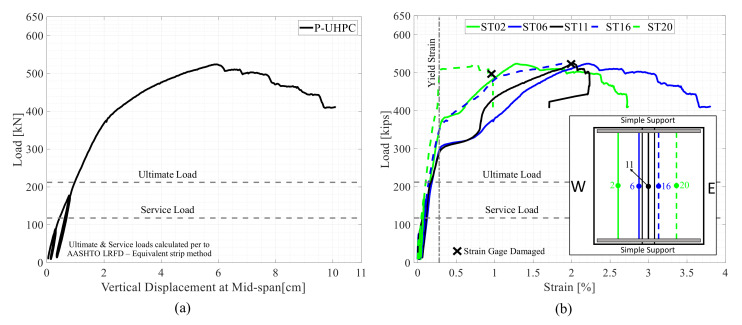
Experimental test results of the reference P-UHPC specimen: (**a**) Load versus mid-span deflection; (**b**) Load versus tensile strains at the middle of the bottom transverse reinforcement.

As mentioned above, the experimental results of a reference specimen with a P-UHPC transverse joint are presented briefly in this section for completeness. This specimen was tested as a part of a comprehensive experimental study which included testing of five large-scale bridge deck specimens with transverse and longitudinal field joints. The main aim of that precedent study was to compare and investigate the structural performance of bridge deck specimens with polymer concrete and P-UHPC field joints [23,24]. The reference specimen had overall planar dimensions of 2.44 m × 2.74 m and a thickness of 20.3 cm. The P-UHPC transverse field joint had a width of 15.24 cm and a diamond-shaped shear key and was located in the middle of the specimen. The details of the P-UHPC joint were proposed based on real bridge practical implementations and the results of many research projects. These typical joint details have been demonstrated to develop sufficient shear and bending capacities to provide integrity between the joined deck panels. The design details of this reference specimen are typical of that of the first specimen which is tested in this study. The specimen was simply supported and loaded at mid-span with a static vertical load. The results of the load versus mid-span deflection relationship of the reference specimen are shown are Figure 2a. Moreover, Figure 2b shows the load versus the reinforcement strain readings of the bottom transverse bars (i.e., main reinforcement) of the P-UHPC specimen. The results shown here are used later for the comparison and assessment of similar specimens with the proposed NP-UHPC transverse joints. The mixing proportions of the P-UHPC mix in comparison with the NP-UHPC mix with 2% steel fibers are shown in Table 3. It is noted that the weight of the dry premix of the NP-UHPC mix represents the sum of the weights of all the dry components including the cement, slag, silica fumes, and sand.

The peak load capacity of the reference specimen was 524.5 kN at which 5.92 cm mid-span vertical displacement was measured. The failure of the specimen was dominated by flexure as it included yielding of the main reinforcement followed by the crushing of concrete at the top of the precast panels. It was observed that no interface cracks or bond slippage had happened up to the peak load capacity of the specimen. Moreover, the specimen had remained essentially elastic in which no reinforcing bars had yielded up to the AASHTO LRFD ultimate load level as shown in Figure 2b.

## 3. Experimental Program

This section provides information regarding the design and structural details of the specimens, fabrication process, experimental test setup, loading methodology, and instrumentation plan.

### 3.1. Specimens Design and Test Matrix

**Figure 3:**
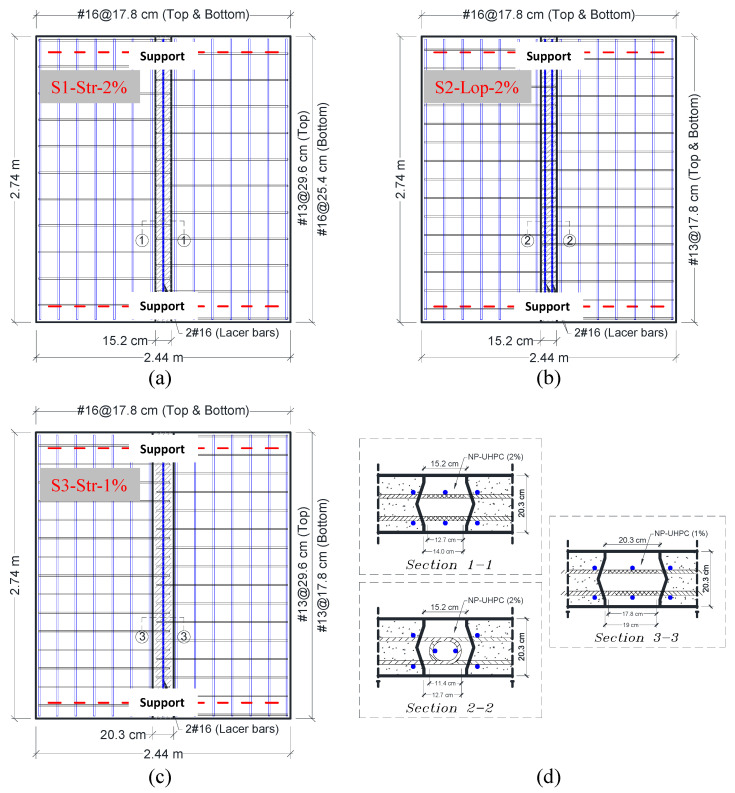
Overall dimensions and structural design details of specimens (**a**) S1-Str-2%; (**b**) S2-Lop-2%; (**c**) S3-Str-1%; (**d**) close-up view of the field joint details.

The experimental program presented in this study included testing three full-scale bridge deck specimens with transverse NP-UHPC field joints. The structural design details of all test specimens are shown in Figure 3. Each specimen consists of two precast deck panels which were cast using conventional concrete with a specified compressive strength of 34.5 MPa. The precast panels were joined together in the transverse direction of the bridge (i.e., perpendicular to the direction of motion of traffic as illustrated in Figure 1 above) using NP-UHPC full-depth field joints. Each panel had a protruded longitudinal reinforcement which was spliced at the field joint location to provide continuity of load transfer across the panels. The experimental program included testing of different parameters, such as different types of reinforcement splices, splice lengths, joint materials, and reinforcement configurations. All specimens had the same overall dimensions and top and bottom transverse reinforcement (i.e., main reinforcement). Non-contact lap-splices were utilized in this study, but they varied in their length and type. Table 4 shows the test matrix and test variables of three tested specimens in addition to the P-UHPC reference specimen which is referred to as “S0”.

The first specimen “S1-Str-2%” had a straight splice with 12.7 cm length, while the second specimen had a loop splice with 11.4 cm length. The loop splice can provide less splice length compared to the straight splice because of the bearing effects at the bend. The use of loop splices inside the field joints was shown to provide better joint performance and better load transfer across the precast panels [24]. A smaller bar diameter (i.e., #13 versus #16) was selected for the longitudinal reinforcement of specimen “S2-Lop-2%” to accommodate the bend diameter requirements in the ACI 318 provisions [30]. The NP-UHPC mixes, which were used in both specimens, have a 2% by volume steel fibers amount.

As mentioned earlier, one of the objectives of this study was to engineer and optimize the materials in the field joints. Hence, an NP-UHPC mix with only half the amount of steel fibers (i.e., 1% by volume) was used for the third specimen “S3-Str-1%”. This was mainly sought to reduce the cost of the material as the steel fibers are the most expensive component in the NP-UHPC composition. Along with that, the authors proposed an increase of the lap splice length of this specimen by 40% to be 17.8 cm. This increase in the splice length was suggested to compensate for the expected lower performance of the NP-UHPC with 1% steel fibers compared to that of the 2% steel fibers which may require a slightly longer development length. The bottom longitudinal reinforcement of specimen “S1-Str-2%” includes #16 bars which were spaced at 25.4 cm. The authors have also suggested using the same amount of steel for the bottom longitudinal reinforcement of the third specimen while using #13 bars at 17.8 cm spacing. The choice of using a smaller bar diameter resulted in narrower spacing between the reinforcement inside the joint which was proved to enhance the field joint performance and overall load distribution over the specimen [24]. In summary, the joint material of the third specimen was optimized to include only half the steel fibers amount. However, the splice length was increased and the spacing of the bottom longitudinal reinforcement was decreased to compensate for the slightly lesser strength of the NP-UHPC. 

The design of the specimens followed the same design provisions of CIP bridge decks in the AASHTO LRFD Bridge Design Specification [31]. This design procedure does not account for the deck discontinuity and the field joint effects. The moment demands were calculated based on the AASHTO equivalent strip method. This method takes into account the largest moment values imposed on the bridge decks from the numerous live loading conditions. The cross-section of the bridge example used to design the test specimens has five steel girders spaced at 3.65 m on the center and a 20.3 cm thick concrete deck slab. Grade 60 reinforcing steel has been used for the reinforcement of the test specimens. The precast deck panels had 2.54 cm and 5.08 cm bottom and top concrete covers, respectively. To facilitate the bridge deck erection and casting of the joints, non-contact lap splices, which were arranged in a staggered formation, were used inside the joint. No adhesive coatings or surface preparation have been used for the surface of the diamond-shaped shear keys. This was done mainly to examine the weakest possible interface between the joint and the panels and to minimize the time and labor required for this task in the real field implementations. All specimens had general outside concrete dimensions of 2.74 m × 2.44 m × 20.3 cm.

### 3.2. Test Setup and Instrumentations

The experimental testing of the test specimens was done at the Earthquake Engineering Laboratory (EEL) at UNR. The test specimens were simply supported over two seat beams and loaded with a vertical static loading from a 980 kN hydraulic actuator at the middle. This led to a three-point bending configuration. The load was applied adjacent to the field joint to produce the highest shear stresses at the interface between the field joint and the precast panel. The typical mode of failure for these types of joints during the service life of bridges are the interface cracks, hence it is essential to investigate the efficiency of the interface bond between the joint and the precast members at the service and ultimate loads. Elastomeric rubber bearings were used on the top of the steel seat beams to allow a zero moment or free rotation at supports. The test setup was not designed to provide fixity at the ends to mimic the real bridge deck case scenario. Thus, the span length of the specimens was set up based on the estimated distance between the bending inflection points. As mentioned earlier, the bridge example had 3.65 m spacing between the main beams. For ideal uniform load distribution over the bridge deck, negative moments were expected near the main beam locations while a positive moment was expected at the middle. The distance between the seat beams was adopted to represent the effective span length or the distance between the bending inflection points where the bending span length was adopted to be 2.44 m. This distance represents almost two-thirds of the 3.65 m main beams spacing from the utilized bridge example. The mid-span vertical load was applied at the edge of the field joint to test the largest possible shear stresses at the interface between the joint and the precast panels.

**Figure 4:**
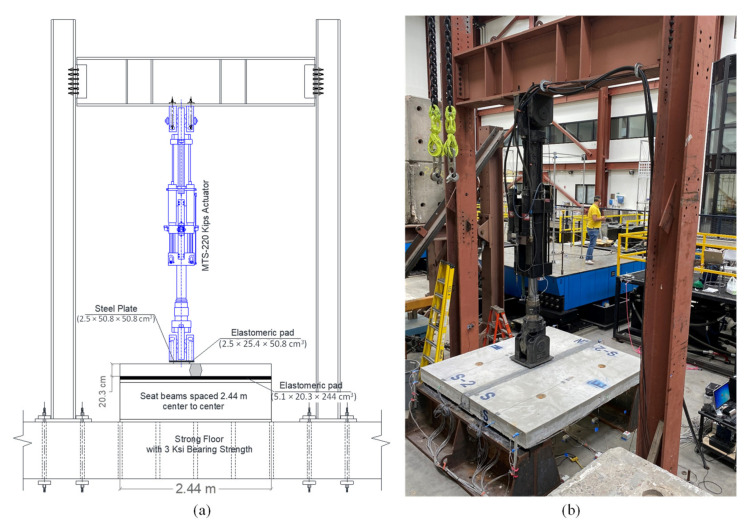
Experimental test setup (**a**) schematic drawing of the test setup; (**b**) photograph of the actual test setup at UNR.

**Figure 5:**
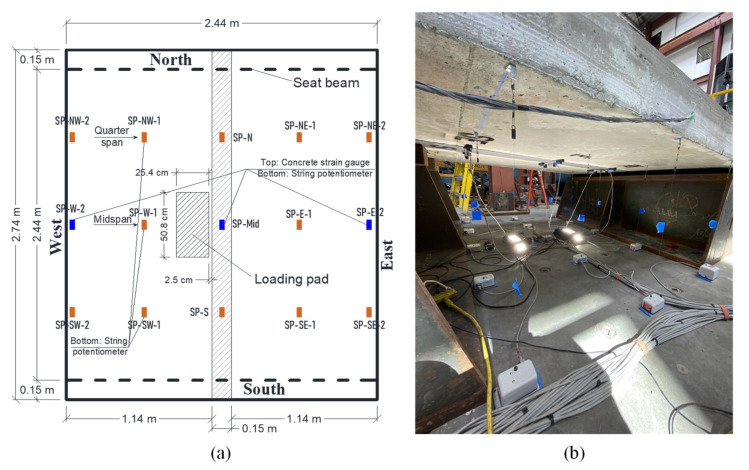
Instrumentation plan (**a**) plan view for the locations of the string potentiometers; (**b**) photograph of the instrumentations.

Figure 4 shows a schematic drawing and a photograph of the test setup used in this study. Several types of instrumentation devices were used to monitor deflections, concrete cracks, and reinforcement and concrete strains at different locations of the specimens and throughout the test. Figure 5 shows some of the instrumentation devices used in this study. Figure 5 also shows the locations of the string potentiometers which were used to measure the deflections of the test specimens.

### 3.3. Loading Protocol

The loading procedure used in the present study, which was also used to test the reference specimen with P-UHPC, i.e., S0, consisted of four cycles of loading and unloading at small load levels. These cycles were followed by a static monotonic loading up to the failure of the specimen. The purpose of establishing these initial cycles was to study the performance of the test specimens under representative service loads. The initial cycles included two 89 kN cycles, then two other 178 kN cycles. Loading and unloading rates of 22.24 kN/min were used during the first four cycles, while the last cycle was controlled by the mid-span deflection at a rate of 1.9 mm/min up to failure.

### 3.4. Fabrication of Test Specimens

**Figure 6:**
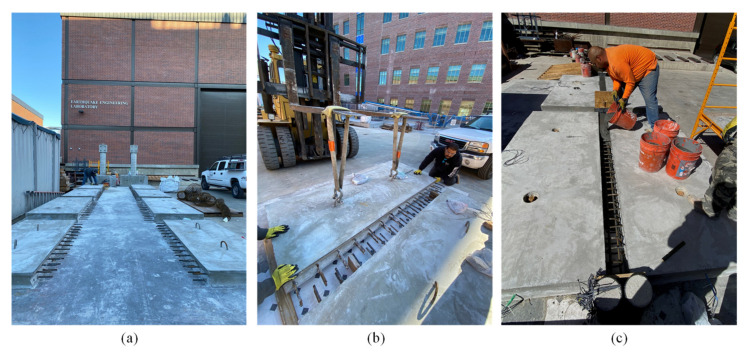
Construction of the test specimens (**a**) fabrication of the deck panels; (**b**) alignment of the deck panels; (**c**) pouring the NP-UHPC inside the field joints.

The construction of the test specimens followed three main phases as illustrated in Figure 6. First, two precast panels were fabricated for each specimen using conventional concrete from a single ready-mix batch. The conventional concrete had a compressive strength of 27.5 MPa at 15 days and 52.4 Mpa at test days. After two weeks, every two panels were aligned together leaving a middle gap to pour the field joints. Finally, the field joints were poured using the NP-UHPC mixes with 2% and 1% steel fibers. The measured compressive strengths at test days of the 2% and 1% NP-UHPC mixes were 146.4 Mpa and 125.7 Mpa, respectively. The P-UHPC reference specimen was fabricated using conventional concrete with 35.8 Mpa compressive strength measured on the day of the test, and the P-UHPC had a compressive strength of 191.7 Mpa on the day of the test.

## 4. Test Results and Discussion

This section shows the experimental test results of the three tested specimens and provides a discussion of the global behavior of the tested specimens in terms of damage progression, modes of failure, and load-deflection relationships. Furthermore, the local behavior of the tested specimens is also reported herein in terms of the load versus the reinforcement and concrete strains. In addition, this section provides evaluation and comparisons of the proposed NP-UHPC systems with the reference P-UHPC specimen to validate the use of the new material for future applications.

### 4.1. Key Results

A brief summary of the key test results is provided in Table 5. The table shows the initial stiffness, load capacities, load at which the reinforcement started to yield and middle deflections of the three NP-UHPC specimens in comparison with the results of the reference P-UHPC specimen. It is known that the bridge decks are designed to remain essentially elastic under the code specified service and ultimate loads. However, the testing of the specimens continued up to failure in order to understand the structural behavior and joint performance at such higher loads and to determine whether the whole system remains intact or the field joint or the joint interface would be the weakest links.

The table shows that the peak load capacities and initial stiffness of the NP-UHPC specimens are higher than that of the reference specimen with P-UHPC. This behavior is attributed to the higher compressive strength of the precast panels of the NP-UHPC specimens, i.e., 52.4 Mpa compared to 35.8 Mpa for specimen S0. The three NP-UHPC specimens had very comparable behaviors. However, specimen S3-Str-1% had slightly less initial stiffness and load capacity because of the lower strength of the NP-UHPC with 1% steel fibers. This resulted in larger deflections at the AASHTO service and ultimate loads. To be able to compare the results of specimen S1-Str-2% with the reference specimen S0, the compressive strength of both specimens was normalized since there is a difference between the compressive strength of the precast panels and the field joints of both specimens. Each specimen was fabricated from two different components (i.e., concrete panels and UHPC joints). The weighted compressive strength of specimen S0, depending on the width and compressive strength of the two components, is 45.54 Mpa. Meanwhile, specimen S1-Str-2% had a weighted compressive strength of 58.27 Mpa. Hence, specimen S1-Str-2% had a 28% higher compressive strength than specimen S0. The 28% higher compressive strength resulted in a 21% increase in the initial stiffness and only a 12% increase in the load capacity of specimen S1-Str-2%.

### 4.2. Global Behavior of Specimens

The global behavior of the tested specimens was evaluated herein in terms of the damage progression, modes of failure and load versus deflection relationships.

#### 4.2.1. Damage Progression and Mode of Failure

**Figure 7:**
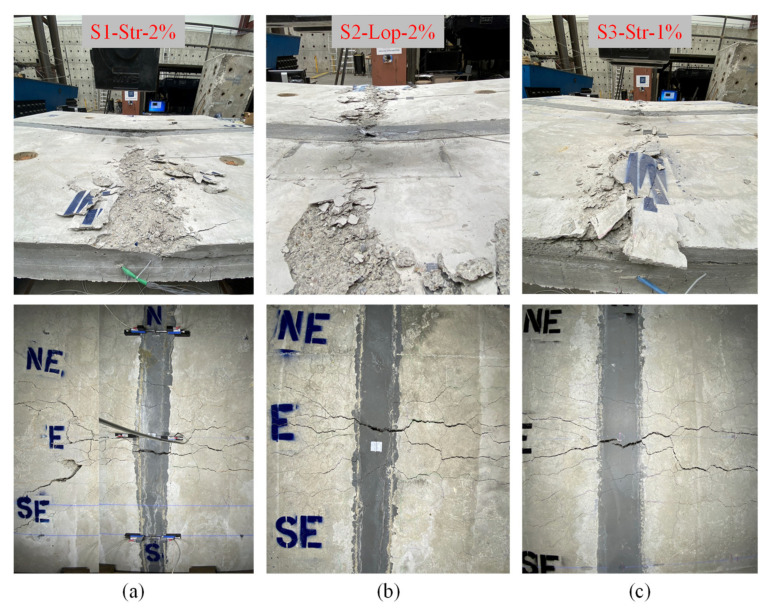
Crack pattern, damage and modes of failure at the top and bottom of specimens (**a**) S1-Str-2%; (**b**) S2-Lop-2%; (**c**) S3-Str-1%.

The observed modes of failure of the test specimens were almost similar, as such flexural members are usually designed to be tension controlled. In this case, the cross-section of the deck specimens is under-reinforced which forces the main reinforcement to yield before the concrete crushing. Hence, the common observed mode of failure for all test specimens is yielding of the bottom transverse reinforcement due to bending followed by crushing of the conventional concrete at the top of the precast panels. Figure 7 shows the damage schemes at the bottom and top sides of the test specimens.

The crushing of concrete was initially observed near the applied load location just before the failure of the specimens at approximately 570 kN. Then, the crushing propagated across the width of the west and east precast panels. It was observed that specimen S1-Str-2% had crushing at the west precast panel only. This was due to the interface crack (see Figure 7a) that happened at the top and bottom of the specimen between the east precast panel and the field joint. This interface crack was the main reason for the interruption of the load transfer path from the loaded west panel to the east panel that resulted in crushing of the west panel only. This damage is similar to the damage of the reference specimen and unlike that of the other two NP-UHPC specimens as there were no interface cracks observed. It is noted that specimens S2-Lop-2% and S3-Str-1% had a denser longitudinal reinforcement which was overlapped inside he joint compared to specimen S1-Str-2%. This denser reinforcement inside the joint may enhance the interface and prevent interface separation between the joint and the precast panels. This note suggests that it is better to use more overlapped reinforcement inside the field joint to avoid interface cracking and to ensure better load distribution across the precast panels. 

Bridge decks are mainly designed to elastically sustain the AASHTO service and ultimate load levels. Thus, it is important to evaluate the damage of the specimens at these specified load levels. At the AASHTO ultimate load, only limited and narrow flexural cracks were observed at the bottom of the precast panels. No flexural or interface cracks were observed on any of the joints at this load level because of the high tensile strength of the NP-UHPC and its higher bond strength with the precast panels. The field joints of specimens S1-Str-2% and S2-Lop-2% were cracked at around 335 kN. The field joint of specimen S3-Str-1% was cracked at earlier loads (i.e., 260 kN) because of the lower tensile strength of the NP-UHPC with 1% steel fibers compared to the 2% steel fibers. On the other hand, the first interface crack was observed at 335, 445, and 400 kN for specimens S1-Str-2%, S2-Lop-2%, and S3-Str-1%, respectively. These interface cracks were located mainly at the middle bottom of the specimens between the field joint and the east precast panel as shown in Figure 7. More importantly, the effect of these cracks was minor and they did not dominate the failure of the specimens as all specimens failed in pure flexural behavior. One more key observation for the tested specimens is that there was no bar slip observed for the lap splices within the joints throughout the test. This means that the proposed lap lengths were adequate to transfer the forces between both precast panels up to failure loads. The test was stopped when a specimen lose 20% of the observed peak load capacity.

#### 4.2.2. Load-Deflection Relationship

**Figure 8:**
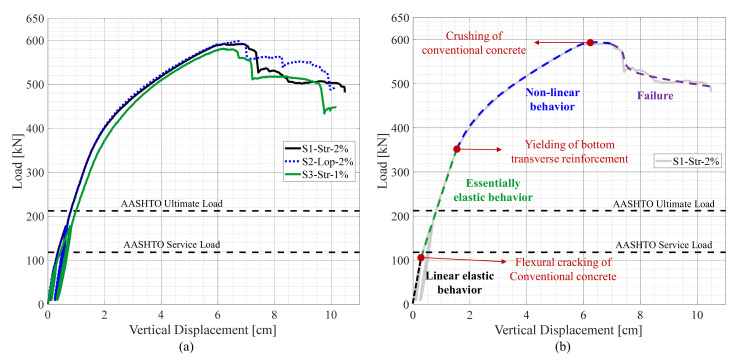
Global Behavior of the NP-UHPC specimens: (**a**) Load versus mid-span deflection relationships of the tested specimens; (**b**) Stages of the flexural behavior of specimen S1-Str-2%.

The global behavior of the test specimens was also evaluated based on the load versus middle deflection relationships. In this section, the load versus deflection relationships of the three test specimens are compared together. The main aim of this section is to investigate the effect of varying the test parameters on the flexural behavior of the specimens. Nonetheless, the overall behavior of the NP-UHPC specimens was also compared with that of the reference P-UHPC, which was provided in Section 2.2. Figure 8a shows the load versus mid-span deflection relationships of the three test specimens. Figure 8b illustrates the different stages of behavior on the load versus deflection relationship of specimen S1-Str-2%. The flexural behavior of the tested specimens is almost similar if not identical. Hence, only the stages of the flexural behavior of specimen S1-Str-2% are shown here as a sample. The AASHTO LRFD limit states, shown in Figure 8, were calculated using the equivalent strip method. This method takes into account the largest possible moment values of the deck slabs with respect to the different loading conditions.

In general, the flexural behavior of the test specimens was very comparable no matter the variation of the test parameters. However, it is shown that specimen S3-Str-1% had slightly softer behavior due to the reduction in stiffness associated with the use of an NP-UHPC mix with only 1% steel fibers. The flexural capacity of the tested specimens had far exceeded the specified AASHTO LRFD ultimate limit state. There are many reasons for this large difference or factor of safety in this case. One reason is that the limit states were calculated based on the nominal steel yielding value of 410 Mpa and nominal concrete compressive strength of 35 Mpa. In reality, the actual steel yielding value was 480 Mpa and the actual compressive strength was 52.4 Mpa. Moreover, at the design stage of the test specimens, a moment reduction factor of 0.9 was used to magnify the moment demand and increase the required bottom transverse reinforcement. Nonetheless, the AASHTO LRFD design procedure does not count for the contribution of the top layer of reinforcement that usually yields to the more required bottom reinforcement. 

The behavior of the tested specimens was similar to that of the P-UHPC specimen and the failure was dominated by flexure of the deck slabs without any major joint or joint interface failure or slippage of rebar lap splices inside the joints. Therefore, the NP-UHPC mixes used in this study with the proposed joint details can be considered as viable solutions for the transverse bridge deck field joints. The proposed NP-UHPC solutions can fulfill the target behavior of the conventional CIP bridge decks in terms of strength and flexure-dominated failure.

The typical flexural behavior of the tested specimens, as shown in Figure 8b, is divided into four main regions. The flexural behavior started with a linear elastic response up to approximately 100 kN in which the applied load was less than the cracking load of the specimens. The second region is defined by the essential elastic behavior in which the conventional concrete was cracked in tension while the reinforcement was not yet yielded. The concrete cracking resulted in a slight decrease in the flexure stiffness compared to the initial stiffness reported in Table 4. The initiation of yielding of the bottom transverse reinforcement was observed at the end of this stage which was associated with flexural and in some cases interface cracking of the field joints. This resulted in a non-linear flexural response of the specimens in which a significant reduction of stiffness was observed. This reduction in flexural stiffness was mainly due to a combination of factors including the aggressive tensile cracking of the concrete, continued yielding of reinforcement, and, to less extent, the interface cracking of the joints. Finally, the failure of the specimens was observed after the crushing of the conventional concrete at the applied load location. This resulted in a global stiffness degradation in which the load capacity of the specimens decreases with increasing the applied vertical displacements.

### 4.3. Local Behavior of Specimens

The local behavior of the tested specimens was evaluated in this section in terms of the load versus the tensile strains of the transverse and longitudinal reinforcement in addition to the load versus the concrete compressive strains. Table 6 shows a summary of the largest recorded tensile strains of the transverse bottom reinforcement (i.e., main reinforcement), in addition to the maximum concrete compressive strains at the AASHTO LRFD service and ultimate loads. The following sections provide more details and discussion about these key results.

#### 4.3.1. Transverse Reinforcement Strains

**Figure 9:**
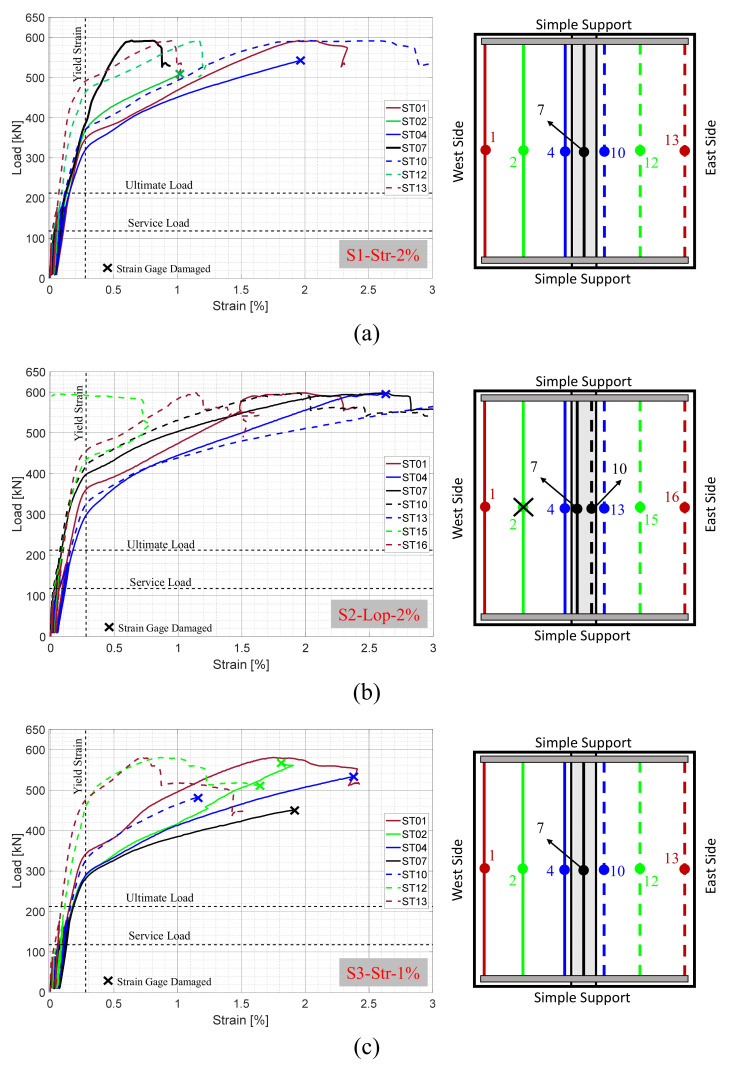
Load versus tensile strains of the bottom transverse reinforcement measured at mid-span of specimens (**a**) S1-Str-2%; (**b**) S2-Lop-2%; (**c**) S3-Str-1%.

The load versus the measured tensile strains of the bottom transverse reinforcement at mid-span (i.e., maximum moment location) are shown in Figure 9. The figure also includes schematic drawings of the test specimens to show the location of the instrumented reinforcing bars and locations of the installed strain gages. It is noted that some of the strain gages were damaged, especially at larger strain levels as expected in typical destructive testing. The damaged strain gages were noted in Figure 9. The main observation in Figure 9 is that the main reinforcing bars have not yielded before reaching the AASHTO LRFD ultimate load. This observation supports the fact that the specimens remained essentially elastic up to and slightly beyond the code allowable limit. This behavior was desirable to confirm that actual behavior exceeds nominal-based calculations, i.e., using the nominal values of 35 Mpa for concrete compressive strength and 400 Mpa for steel yielding strength of 400 Mpa to calculate the AASHTO ultimate load. The actual strength values usually surpass these nominal values. Another reason for confirming the factor of safety belongs to the use of a reduction factor of 0.9 during the design stage, which increases the moment demand on the cross-section and consequently increases the required reinforcement. Nevertheless, the observed behavior verifies the acceptable performance of the NP-UHPC mixes as closure joint materials for the field joints of precast bridge decks.

The yielding was initially observed either in the reinforcing bars which were located inside the field joints or adjacent to the west side of the joints. The first yield in the main reinforcing bars was observed at approximately 320, 298, and 281 kN for specimens S1-Str-2%, S2-Lop-2% and S3-Str-1%, respectively. The onset of yielding was followed by a sequence of yielding of the adjacent bottom transverse reinforcement. The excessive yielding of reinforcement resulted in global softening of specimens and consequently change of the flexural behavior to the non-linear response (see Figure 8b). It is also noted that the reinforcing bars which were located on the west precast panel were more stressed than the bars on the east side panel. This is attributed to the eccentricity of loading in the east-west direction as the load was applied on the west side of the field joint (see Figure 4). The two middle bars which were located inside the field joint of specimen S2-Lop-2% are usually called lacer bars. These bars are usually used to connect the inner tip of the loop splices (see Figure 3) to enhance the bearing reactions of the splices and increase ductility. As a result of the location of these lacer bars which were not located at the outermost surface of the joint, the tensile strains of these bars were found to be slightly lower than the other adjacent bars outside the joint.

#### 4.3.2. Longitudinal Reinforcement Strains

**Figure 10:**
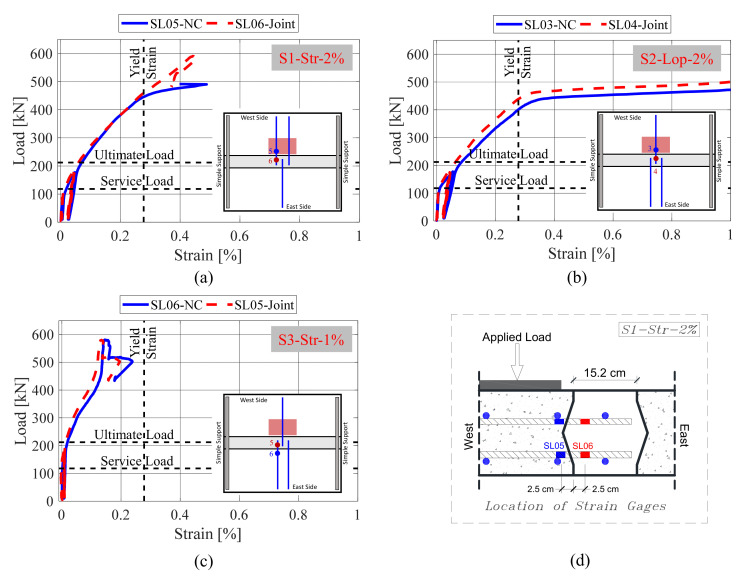
Load versus tensile strains of selected bottom longitudinal reinforcement of specimens (**a**) S1-Str-2%; (**b**) S2-Lop-2%; and (**c**) S3-Str-1%; (**d**) close-up view for the locations of the strain gages.

The previous section covered the tensile strains of the bottom transverse reinforcement (i.e., main flexural reinforcement). Thus, this complementary section focuses on the tensile strains of the longitudinal reinforcement (i.e., secondary flexural reinforcement) which were overlapped inside the joints. The strain gages were attached to the longitudinal bars near the two sides of the interface between the field joint and the precast panels as shown in Figure 10. The location of the strain gages was chosen based on two main reasons. First, the strain readings from both strain gages were intended to be compared together to anticipate if there is slippage of the reinforcement splices inside the joint. Second, the strain readings of the strain gages which were installed inside the joints (marked in red in Figure 10) were intended to be verified with the yield strain to determine if the development length was sufficient to yield the bars inside the joint. The two ways of verification may indicate if the proposed joint details and the utilized NP-UHPC mixes are sufficient to transfer the load from the west to the east precast panels. In this case, the proposed multi-component deck system can be considered equivalent to the monolithic CIP bridge decks. Figure 10 shows the results of the load versus the tensile strains of selected bottom longitudinal reinforcement. Figure 10 shows the maximum strain values of the longitudinal bars. It is noted that many of the strain gages were damaged during the construction of the specimens and field joints. Hence, the maximum strain results of the longitudinal bars of specimen S3-Str-1% were not shown in Figure 10.

It was observed that the measured tensile strains of inside and outside the field joint were almost typical with no signs of bar slippage occurring up to the peak load. Nonetheless, the measured strain values of the longitudinal bars inside the joint for specimens S1-Str-2% and S2-Lop-2% exceeded the yield strain of reinforcement. This confirms that the proposed overlap length was sufficient to develop the yielding of reinforcement inside the joints. At the AASHTO ultimate load, the measured tensile strain values were typically far below the yield strain. This suggests that the demand on the field joints within the AASHTO design loads does not dictate the need for the full development length of the bars inside the joints. However, the use of the full development length may be required if the deck slabs were to be subjected to more demand loads than the specified AASHTO ultimate loads.

#### 4.3.3. Concrete Compressive Strains

**Figure 11:**
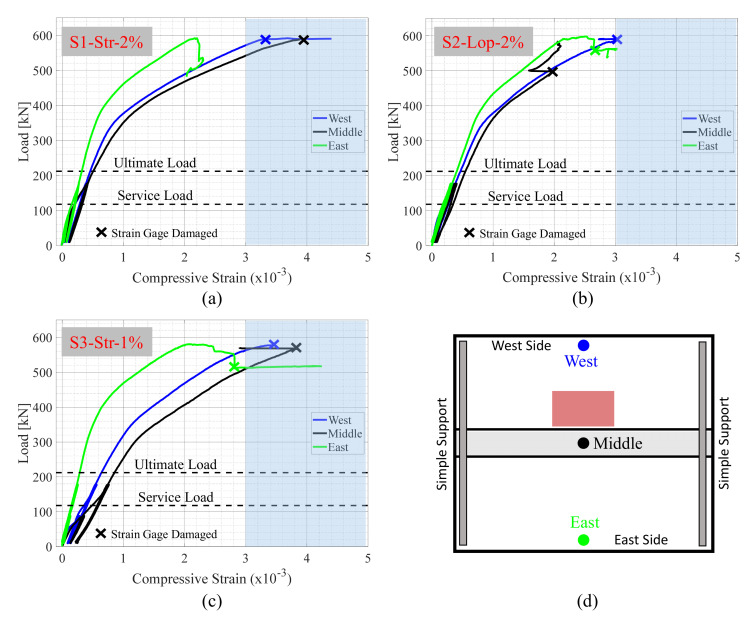
Load versus concrete compressive strain measured at mid-span of specimens (**a**) S1-Str-2%; (**b**) S2-Lop-2%; and (**c**) S3-Str-1%.; (**d**) sketch of the test specimens with the locations of the concrete strain gages.

The previous section focused on the tensile strains of the transverse and longitudinal reinforcement. Nonetheless, the load versus compressive strain readings of the conventional concrete and NP-UHPC are presented in this section (see Figure 11) for completeness. The concrete strain gages were placed at mid-span (maximum moment location) to measure the maximum compressive strength at the extreme concrete compression fibers of the precast panels and the field joint. The measured strain values were compared with the strains at which crushing of the conventional concrete and NP-UHPC were expected to happen. The crushing of the unconfined conventional concrete and NP-UHPC were expected to happen at compressive strains of approximately 0.003 and 0.005, respectively as per previous studies [26,30,31]. Hence, Figure 11 includes shaded areas, highlighted in blue color, to show the boundaries of the concrete crushing strain values. It is noted that some of the concrete strain gages were damaged during the test, especially at higher load values. Figure 11 shows when the concrete strain gages were damaged.

As mentioned earlier, crushing of concrete was observed slightly before the failure of the test specimens. The same observation can also be confirmed from the results shown in Figure 11. In the figure, the compressive strain readings indicated that most of the concrete strains are slightly more than 0.003 at the peak loads of the test specimens. The crushing of concrete was observed on the east and west precast panels, and similarly on the field joints. This behavior was slightly different from that of specimen S1-Str-2% as no crushing was observed in the east precast panel. The compressive strain values of specimen S1-Str-2%, which were measured at the east precast panel, were smaller than the crushing strain values. This is attributed to the interface crack between the field joint and the east precast panel that was the main reason for interrupting the load path from the applied load location to the east panel. The concrete compressive strains of the east precast panel are slightly lower than the strains measured at the west panel and the field joint. This was expected because of the eccentricity of loading in the east-west direction. It was observed that specimen S2-Lop-2% has a slightly better strain distribution over the cross-section when compared to the other specimens. This indicates that the use of a loop splice instead of the straight lap splice enhanced the load distribution over the specimen cross-section. On the other hand, the dispersion of the concrete strains values of specimen S3-Str-1% may indicate a less favorable load distribution across the precast panels because of the use of an NP-UHPC mix with only 1% steel fibers.

## 5. Conclusions

This paper summarizes the main results of the comprehensive full-scale experimental testing of representative precast bridge deck panels with NP-UHPC transverse field joints. The presented scope of work was part of a bigger research project that aims at developing and promoting NP-UHPC mixes and demonstrating their viability for various ABC applications, such as field joints of precast bridge decks. In this study, two NP-UHPC mixes with ingredients sourced from the western states (Nevada and California) were used as a closure joint material. The paper provides a brief discussion about the mix design and main mechanical properties of the utilized NP-UHPC mixes for completeness along with results of a similar specimen with P-UHPC transverse field joints for comparison purposes. The experimental program included testing three full-scale bridge deck specimens with transverse NP-UHPC joints. The test parameters included different joint splice details, joint widths, closure joint materials, and longitudinal reinforcement configurations. With the detailed discussion of the structural performance of the test specimens in terms of both global and local behaviors and comparison with reference P-UHPC specimen, the following observations and conclusions can be drawn:

- In general, the structural behavior and joint performance of the precast bridge decks with full-depth transverse NP-UHPC field joints are proof-tested and demonstrated to be acceptable and viable for ABC. As the proposed field joints adequately maintained the load distribution capabilities along the specimen’s cross-section up to the AASHTO ultimate loading without any major cracking or interface failure.
- The global and local behaviors of the test specimens with transverse NP-UHPC are shown to be very comparable to that of representative specimens of the readily implemented and acceptable practice of using commercial or proprietary UHPC mixes.
- All the test specimens had flexure-dominated failures in which yielding of reinforcement was observed before the concrete crushing and failure. As the yielding of the bottom transverse bars was observed at approximately 300 kN, while the crushing of concrete was observed approximately at 580 kN. In all tests, the NP-UHPC joints were confirmed to remain intact up to the full structural system failure level, i.e., NP-UHPC joints are not the weakest links in integrated deck systems.
- The initial stiffness and load capacities of the deck systems with NP-UHPC, which use a 2% by volume steel fibers amount, are slightly greater than that of the deck systems with NP-UHPC joints with only 1% steel fibers. For example, the peak load and initial stiffness of specimen S1-Str-2% were 592 kN and 510.6 kN/cm, respectively compared to 581 kN and 460.1 kN/cm for specimen S3-Str-1%. However, this slight reduction in stiffness does not have any implications for meeting the desired code limit states.
- The proposed deck systems with NP-UHPC transverse joints are able to fulfill the AASHTO LRFD service and ultimate load requirements without any major damage, splice slippage or interface cracking. In the conducted tests, only some interface cracks were observed at later loading stages which did not affect the failure of the specimens.
- The test specimens remained essentially elastic up to the AASHTO LRFD ultimate load in which no yielding of reinforcement was observed. The initiation of yielding was observed at the bottom transverse reinforcement at approximately 300 kN. The tensile strains of the longitudinal splices inside the joint indicated that the proposed overlap lengths were sufficient to yield the reinforcement inside the joint.
- The compressive strength of the conventional concrete and NP-UHPC showed that concrete crushing took place just before the failure of the specimens and after the yielding of the reinforcement (i.e., tension-controlled behavior is confirmed). The measured concrete strains indicate that the loop splices enhanced the load distribution across the specimen’s cross-section. Only the use of an NP-UHPC mix with 1% steel fibers would have a slightly less favorable load distribution, but with no adverse effects on meeting design requirements.
- Overall, the proposed NP-UHPC mixes with 1% and 2% steel fibers can be effectively used as closure joint materials for transverse field joints in precast bridge decks as they were able to provide full development for the reinforcement inside the joint and the provided adequate interface bond up to the AASHTO LRFD ultimate load.
- The performance of this material in field joints is very comparable to commercial/proprietary UHPC mixes as the peak load capacity of the NP-UHPC specimen was 592 kN compared to 524.5 kN for a similar P-UHPC reference specimen. Meanwhile, the NP-UHPC is cheaper than the P-UHPC where the typical P-UHPC and NP-UHPC material cost is estimated to be around $3300 and $1300 per cubic meter, respectively.
- The proposed transverse joint details and NP-UHPC materials are also able to provide equivalent behavior to monolithic CIP deck system as rendered from the observed failure mode, load distribution, or strain demands as the specimens remained elastic up to the AASHTO LRFD ultimate loads and the failure mode was mainly dominated by flexure of the specimens.

## Figures and Tables

**Table 1:** Mixing Proportions and Material Sources of the NP-UHPC Mixes.

Material	1% Steel Fiber Mix Quantity, kg/m^3^	2% Steel Fiber Mix Quantity, kg/m^3^	Material Supplier
Cement	707	700	Type I/II Nevada Cement, Reno, NV, USA
Slag	354	350	Lehigh, Sacramento, CA, USA
Silica Fumes	118	117	BASF (Master Life SF 100)
Water	236	233	Potable Water
w/b	0.2	0.2	
Sand	1179	1166	Crushed Aggregate Sand, Martin Marietta Sparks, Sparks, NV, USA
Steel Fibers	79	151	Bekaert (Dramix OL 13/0.2)
Superplasticizer	13.5	13.5	BASF (Glenium 7920)

**Table 2:** Main Mechanical Properties Tested at 28 days for the NP-UHPC Mixes.

Property	1% Steel Fibers Mix	2% Steel Fibers Mix	Standard Test Method
Compressive Strength, MPa	128.9	114.9	Modified ASTM C39 [28]
Flexural Strength, MPa	14.3	20.7	ASTM C1609 [29]
Direct Tensile Strength, MPa	4.83	5.64	N/A

**Table 3:** Mixing Proportions of the P-UHPC and NP-UHPC Components.

Material	P-UHPC Weight (kg/m^3^)	Percentage by Weight (%)	NP-UHPC Weight (kg/m^3^)	Percentage by Weight (%)
Dry premix	2195	87.6	2333	85.5
Water or ice	130	5.0	233	8.5
Superplasticizer	30	1.2	13.5	0.5
Steel fibers (2% by volume)	156	6.2	151	5.5

**Table 4:** Experimental Test Matrix and Specimen Design Details.

Specimen Name	Transverse Reinforcement		Longitudinal Reinforcement		Field Joint Material	Lap Splice Type	Lap Splice Length (cm)
	Top	Bottom	Top	Bottom			
S0 (reference)	#16 @ 17.8cm	#16 @ 17.8cm	#13 @ 38.1 cm	#16 @ 25.4 cm	P-UHPC 2%	Straight	12.7
S1-Str-2%			#13 @ 29.6 cm	#16 @ 25.4 cm	NP-UHPC 2%	Straight	12.7
S2-Lop-2%			#13 @ 17.8 cm	#13 @ 17.8 cm	NP-UHPC 2%	Loop	11.4
S3-Str-1%			#13 @ 29.6 cm	#13 @ 17.8 cm	NP-UHPC 1%	Straight	17.8

Abbreviations; Str: Straight splice, Lop: Loop splice.

**Table 5:** Summary of Key Experimental Test Results.

Specimen Name	Peak Load (kN)	Load @ 1st Yield (kN)	Load @ 1st Interface Crack (kN)	Mid-span Deflection (cm)			Initial Stiffness, (kN/cm)
				Peak Load	Service Load	Ultimate Load	
S0	524.5	≈290	520	5.920	0.444	0.975	420.3
S1-Str-2%	592.0	≈320	335	6.217	0.349	0.811	510.6
S2-Lop-2%	598.1	≈298	445	6.697	0.345	0.805	545.3
S3-Str-1%	581.0	≈281	400	6.183	0.437	0.992	460.1

**Table 6:** Summary of the Maximum Tensile Reinforcement Strains and Cncrete Compressive Strains at the AASHTO LRFD Service and Ultimate Loads.

Specimen Name	Maximum Tensile Strain @ Bottom Transverse Reinforcement (%)		Maximum Concrete Compressive Strains (×10^−3^)			
	Service Load	Ultimate Load	Precast Panels		NP-UHPC Joint	
			Service Load	Ultimate Load	Service Load	Ultimate Load
S1-Str-2%	0.068	0.154	0.30	0.65	0.29	0.72
S2-Lop-2%	0.069	0.174	0.38	0.73	0.39	0.88
S3-Str-1%	0.083	0.183	0.36	0.64	0.49	0.86

## References

[1] American Road and Transportation Builders Association (ARTBA) (2020). 2020 Bridge Report.

[2] Zhu P., Ma Z.J., Cao Q., French C.E. (2012). Fatigue Evaluation of Transverse U-Bar Joint Details for Accelerated Bridge Construction. J. Bridg. Eng..

[3] Li L., Jiang Z. (2016). Flexural Behavior and Strut-and-tie Model of Joints with headed bar details Connecting Precast Members. Perspect. Sci..

[4] Badie S.S., Tadros M.K. (2008). Full-depth precast concrete bridge deck panel systems. Transp. Res. Board.

[5] Verger-Leboeuf S., Charron J.-P., Massicotte B. (2017). Design and Behavior of UHPFRC Field-Cast Transverse Connections between Precast Bridge Deck Elements. J. Bridg. Eng..

[6] Graybeal B.A. (2010). Behavior of Field-Cast Ultra-High Performance Concrete Bridge Deck Connections under Cyclic and Static Structural Loading.

[7] French C.E., Shield C.K., Klaseus D., Smith M., Eriksson W., Ma Z.J., Zhu P., Lewis S., Chapman C.E. (2011). Cast-in-Place Concrete Connections for Precast Deck Systems.

[8] Perry V., Krisciunas R., Stofko B. (2014). Mackenzie River twin bridges:North America’s largest field-cast ultra-high performance concrete connections project. PCI J..

[9] Sritharan S., Aaleti S., Garder J., Bierwagen D., Abu-Hawash A. (2012). Use of Ultra-High Performance Concrete in Bridge Design.

[10] Qiao P., Zhou Z., Allena S. (2016). Developing Connections for Longitudinal Joints between Deck Bulb Tees-Development of UHPC Mixes with Local Materials.

[11] Graybeal B. (2013). Development of Non-Proprietary Ultra-High Performance Concrete for Use in the Highway Bridge Sector.

[12] Aboukifa M., Moustafa M.A., Saiidi M.S. (2020). Seismic Response of Precast Columns with Non-Proprietary UHPC-Filled Ducts ABC Connections.

[13] Aboukifa M., Moustafa M.A., Saiidi M.S. (2021). Seismic response of precast bridge columns with composite non-proprietary UHPC filled ducts ABC connections. Compos. Struct..

[14] Berry M., Snidarich R., Wood C. (2017). Development of Non-Proprietary Ultra-High Performance Concrete.

[15] Alsalman A., Dang C., Hale W.M. (2017). Development of ultra-high performance concrete with locally available materials. Constr. Build. Mater..

[16] Wille K., Naaman A.E., Parra-Montesinos G.J. (2011). Ultra-High Performance Concrete with Compressive Strength Exceeding 150 MPa (22 ksi): A Simpler Way. ACI Mater. J..

[17] Subedi D., Moustafa M.A., Saiidi M.S. (2019). Non-Proprietary UHPC for Anchorage of Large Diameter Column Bars in Grouted Ducts.

[18] Tadros M.K., Gee D., Asaad M., Lawler J. (2020). Ultra-High-Performance Concrete: A Game Changer in the Precast Concrete Industry. PCI J..

[19] Kim H., Moon B., Hu X., Lee H., Ryu G.-S., Koh K.-T., Joh C., Kim B.-S., Keierleber B. (2021). Construction and Performance Monitoring of Innovative Ultra-High-Performance Concrete Bridge. Infrastructures.

[20] Abokifa M., Moustafa M.A. (2021). Development of Non-Proprietary UHPC Mix: Application to Deck Panel Joints.

[21] Shahrokhinasab E., Garber D. (2021). Development of “ABC-UTC Non-Proprietary UHPC” Mix, Final Report # ABC-UTC-2016-C2-FIU01-Final.

[22] Looney T., McDaniel A., Volz J., Floyd R. (2019). Development and Characterization of Ultra-High Performance Concrete With Slag Cement for Use as Bridge Joint Material.

[23] Abokifa M., Moustafa M.A. (2020). Experimental behavior of poly methyl methacrylate polymer concrete for bridge deck bulb tee girders longitudinal field joints. Constr. Build. Mater..

[24] Abokifa M., Moustafa M.A., Itani A.M. (2020). More Choices for Connecting Prefabricated Bridge Deck Elements (No. ABC-UTC-2016-C1-UNR03-Final).

[25] Abokifa M., Moustafa M.A. (2021). Mechanical characterization and material variability effects of emerging non-proprietary UHPC mixes for accelerated bridge construction field joints. Constr. Build. Mater..

[26] Abokifa M., Moustafa M.A. (2021). Full-scale testing of non-proprietary ultra-high performance concrete for deck bulb tee longitudinal field joints. Eng. Struct..

[27] California Department of Transportation (2015). Notice to Bidders and Special Provision.

[28] ASTM International (2012). Standard Test Method for Compressive Strength of Cylindrical Concrete Specimens, ASTM C39.

[29] American Society of Testing and Materials (2007). ASTM C1609/C1609M-07. Standard Test Method for Flexural Performance of Fiber-Reinforced Concrete (Using Beam with Third-Point Loading).

[30] ACI Committee (2008). Building Code Requirements for Structural Concrete (ACI 318-08) and Commentary.

[31] AASHTO (American Association of State Highway and Transportation Officials) (2014). AAHSTO LRFD Bridge Design Specifications.

